# Increased CD4+/CD8+ Double-Positive T Cells in Chronic Chagasic Patients

**DOI:** 10.1371/journal.pntd.0001294

**Published:** 2011-08-23

**Authors:** Nicolas A. Giraldo, Natalia I. Bolaños, Adriana Cuellar, Fanny Guzman, Ana Maria Uribe, Astrid Bedoya, Natalia Olaya, Zulma M. Cucunubá, Nubia Roa, Fernando Rosas, Víctor Velasco, Concepción J. Puerta, John M. González

**Affiliations:** 1 Grupo de Ciencias Básicas Médicas, Facultad de Medicina, Universidad de los Andes, Bogotá, Colombia; 2 Grupo de Inmunobiología y Biología Celular, Facultad de Ciencias, Pontificia Universidad Javeriana, Bogotá, Colombia; 3 Núcleo Biotecnología Curauma (NBC), Pontificia Universidad Católica de Valparaíso, Valparaiso, Chile; 4 Facultad de Medicina, Pontificia Universidad Javeriana, Bogotá, Colombia; 5 Grupo de Infección y Cáncer, Facultad de Medicina, Universidad de Antioquia, Medellín, Colombia; 6 Instituto de Patología, Facultad de Medicina, Universidad de Antioquia, Medellín, Colombia; 7 Grupo de Parasitología, Instituto Nacional de Salud, Bogotá, Colombia; 8 Clínica Abood Shaio, Bogotá, Colombia; 9 Laboratorio de Parasitología Molecular, Departamento de Microbiología, Facultad de Ciencias, Pontificia Universidad Javeriana, Bogotá, Colombia; Federal University of São Paulo, Brazil

## Abstract

**Background:**

CD4+/CD8+ double positive (DP) T cells have been described in healthy individuals as well as in patients with autoimmune and chronic infectious diseases. In chronic viral infections, this cell subset has effector memory phenotype and displays antigen specificity. No previous studies of double positive T cells in parasite infections have been carried out.

**Methodology/Principal Findings:**

Seventeen chronic chagasic patients (7 asymptomatic and 10 symptomatic) and 24 non-infected donors, including 12 healthy and 12 with non-chagasic cardiomyopathy donors were analyzed. Peripheral blood was stained for CD3, CD4, CD8, HLA-DR and CD38, and lymphocytes for intracellular perforin. Antigen specificity was assessed using HLA*A2 tetramers loaded with *T.* cruzi K1 or influenza virus epitopes. Surface expression of CD107 and intracellular IFN-γ production were determined in K1-specific DP T cells from 11 chagasic donors. Heart tissue from a chronic chagasic patient was stained for both CD8 and CD4 by immunochemistry. Chagasic patients showed higher frequencies of DP T cells (2.1%±0.9) compared with healthy (1.1%±0.5) and non-chagasic cardiomyopathy (1.2%±0.4) donors. DP T cells from Chagasic patients also expressed more HLA-DR, CD38 and perforin and had higher frequencies of *T. cruzi* K1-specific cells. IFN-γ production in K1-specific cells was higher in asymptomatic patients after polyclonal stimulation, while these cells tended to degranulate more in symptomatic donors. Immunochemistry revealed that double positive T cells infiltrate the cardiac tissue of a chagasic donor.

**Conclusions:**

Chagasic patients have higher percentages of circulating double positive T cells expressing activation markers, potential effector molecules and greater class I antigenic specificity against *T. cruzi*. Although K1 tetramer positive DP T cell produced little IFN-γ, they displayed degranulation activity that was increased in symptomatic patients. Moreover, K1-specific DP T cells can migrate to the heart tissue.

## Author Summary

Chagas disease, produced by the blood parasite *Trypanosoma cruzi*, is considered a public health problem in Central and South America. Non sterile immunity can be achieved after acute infection. Parasite persistence can induce tissue damage in nearly 20% to 30% of chronically infected individuals. Indeed, chagasic cardiomyopathy is one of the consequences of the chronic infection. Antigen persistence and dysfunctional cellular immune response have been implicated in *T. cruzi* pathogenesis. Here, a higher frequency of circulating CD4+/CD8+ double positive T cells in chronic chagasic patients is reported as compared with non infected donors, including those with a non-chagasic cardiomyopathy. This cell subset also expressed more activation markers and stored more intracellular perforin. We have previously reported that CD8+ T cells from *T. cruzi* infected donors recognized the HLA-A*0201 restricted K1-peptide derived from the KMP-11 protein. Here, double positive T cells displayed higher percentages of recognition for the K1 peptide than single CD8+ T cells. These cells produce little IFN-γ, but display degranulation activity that was increased in the symptomatic group. Finally, double positive T cells can be localized in the heart tissue from a chronic chagasic donor.

## Introduction

Expression of either CD4 or CD8 on mature peripheral CD3+ T cells is considered to be a mutually exclusive event as a result of the thymic selection, reflecting the specific functions of each major T cell subpopulation. Contrary to this conventional dichotomy, circulating CD4+/CD8+ double positive (DP) T cells have been identified in human peripheral blood and represents between 1 and 3% of the total T lymphocytes population [1]. The role of these DP T cells in health and disease is still under investigation.

Some healthy individuals can display a significant proportion of DP T cells in peripheral blood. Furthermore, previous evidence has suggested that their frequency in blood can increase or they can be localized in specific tissues during several inflammatory diseases [2]–[8], including: a) chronic viral diseases, like EBV infectious mononucleosis [3] and HIV [4]; b) autoimmune pathologies characterized by chronic lymphocytes activation, such as autoimmune thyroiditis [5], myasthenia gravis [6] and systemic sclerosis/scleroderma [7]; c) allergy i.e. atopic dermatitis [8] and d) some neoplasias [3], [9].

Based on the intensity of CD4 and CD8 expression by flow cytometry, two subsets of DP T cells have been defined: CD4^dim^/CD8^bright^ and CD4^bright^/CD8^dim^ lymphocytes [3]. Previous studies of their phenotypic characteristics have shown that the majority of these cells have memory phenotype (CD45RO+). They are also more differentiated than mono-positive T cells, based on its low level of expression of CD27, and they frequently produced either intracellular granzyme B or perforin [10], [11]. Functional assays showed that during chronic viral infections, DP T cells secrete cytokines, such as IFN-γ, in response to cognate class I restricted antigens [10]. All these results suggest that, although small, DP T cells constitute a highly differentiated memory subpopulation acting in the adaptive immune response against infectious agents [1]–[4], [10], [11].

As this cell population is expanded in several viral and immunological mediated chronic diseases, it seems plausible that DP T cells can contribute to the immune response against chronic parasitic infections. The goal of this study was to determine the frequency, phenotype and effector potential of circulating DP T cells in patients chronically infected with *T. cruzi*, the etiological agent of Chagas disease. This hemoflagellate parasite, which is found in Central and South America, is transmitted by vectors of the *Reduviidae* family and produces both an acute and a chronic phase [12], [13]. Prevalence of Chagas diseases in Colombia is calculated to be 1'300,000, while 3'500,000 people are at risk of contracting the infection, representing a potential public health problem [13], [14]. After *T. cruzi* inoculation, during the acute phase, the parasite extracellular stages are found in peripheral blood and infected individuals develop constitutive symptoms. Activation of the immune system allows parasite control, although it is not completely eliminated. Parasites invade cells such as monocytes/macrophages, dendritic cells, fibroblast and myocardiocytes, shedding their flagella and becoming intracellular [15]. By mechanisms not clearly understood, parasites persist leading to the chronic phase of the disease. Most of the infected individuals will remain asymptomatic for several years, being classified as indeterminate. Those patients are usually detected by routine serological tests in blood banks. Nearly 20% to 30% of the chronic infected patients will develop tissue damage, being Chagasic cardiomyopathy or digestive disease the most common pathologies [12], [13]. What mediates the tissue damage in Chagas is not well understood, but some evidence suggests that parasite persistence and dysfunctional cellular immune response could contribute to this process [12], [15]. During the infection, antibodies elicited against *T. cruzi* antigens help to control blood circulating parasite [16], [17] and specific CD4+ and CD8+ T cells act against intracellular forms [18], [19]. Mice models demonstrated that T cells response seemed to be crucial for parasite control [20]. Interestingly, acute infection with *T. cruzi* in mice showed a large increase of CD4+/CD8+ DP T cells in their subcutaneous lymph nodes [21]. Given the characteristics of the DP T cells (memory phenotype and presence of cytotoxic granules), it is of special interest to determine their functional characteristics in chronic chagasic patients.

## Methods

### Ethics statement

Research protocols and informed consents were approved by the Ethical Committees of the Universidad de los Andes (039-2009), Pontificia Universidad Javeriana (01-2010) and the Fundación Abood Shaio (134-2010), Bogotá, Colombia, following the national regulations and the Declaration of Helsinki.

### Human donors

Forty-one volunteers who signed the informed consent were enrolled in this study and divided into three groups. The first group included 17 chronic chagasic patients with positive immunofluorescence indirect assay (IFI) and ELISA tests. It was composed of 13 females and 4 males with ages ranging from 36 to 67 years old, recruited at the Fundación Abood Shaio (Bogotá, Colombia) or at Instituto Nacional de Salud; and classified according to the Kuschnir grading system [22]. Seven individuals were classified as indeterminate chagasic patients with no significant findings during clinical assessment (G0 or asymptomatic) and 10 as cardiac chronic chagasic patients (symptomatic) graded as follows: 3 with only abnormal ECG results (G1), 5 with abnormal ECG results and cardiac enlargement (G2) and 2 with abnormal ECG results, cardiac enlargement and clinical signs of heart failure (G3). The second group included 12 donors with cardiomyopathy of non-infectious etiology, including 8 females and 4 males with ages ranging from 28 to 80 years old, and whose anti-*T. cruzi* antibodies were negative. In this group, cardiomyopathy etiology was: ischemic cardiomyopathy (n = 6), systemic arterial hypertension (n = 4) and idiopathic dilated cardiomyopathy (n = 2). This group of patients was recruited at the Department of Cardiology, Hospital Universitario San Ignacio, Bogotá, Colombia. The third group included 12 uninfected donors who never lived in a Chagas endemic area, and with negative IFI and ELISA tests. They were 9 females and 3 males with ages ranging from 34 to 68 years old. The characteristics of the three groups, denoted as chagasic patients (CP), non-chagasic cardiomyopathy (NCC) and healthy controls (HC), including main clinical, electrocardiogram and echocardiography findings, are resumed in Table 1.

**Table 1:** Characteristics of the studied individuals.

Characteristics				Groups		
	CP (n = 17)				NCC (n = 12)	HC (n = 12)
Age (years), mean ± SD*	52.8±10.2				62.4±15.2	51.7±11.2
Age range	36–67				28–80	34–68
Female No (%)**	13 (76.5)				8 (66.7)	9 (75.0)

	Asymptomatic	Symptomatic				
	G0 (n = 7)	G1 (n = 3)	G2 (n = 5)	G3 (n = 2)		
LVEF % Mean ± SD	55.0±5.8	58.7±11.8	50.5±17	35.0±7.1	43.8±13.7	-
Atrial fibrillation	-	1	-	-	2	-
Anterior myocardial infarction	-	-	-	-	1	-
Atrioventricular block-I	1	-	1	-	-	-
Atrioventricular block	-	-	-	2	-	-
Right bundle branch block	-	2	2	-	1	-
Sinus bradycardia	1	-	2	-	4	-
Normal ECG	5	-	-	-	-	-

Groups: CP chagasic patients, NCC non-chagasic cardiomyopathy donors and HC healthy controls. Atrioventricular block-I (first degree), LVEF left ventricular ejection fraction.

*p value: 0.08 and.

**p value: 0.89.

### Blood sample and cell surface phenotype

Blood samples were obtained from each donor using EDTA vacuntainer tubes. Total blood (100 µl/tube) was stained with anti-CD3 APC (clone SK7), anti-CD4 PerCP (SK3), anti-CD8 PE (RPA-T8), anti-HLA-DR PE-Cy7 (L243) and anti-CD38 FITC (HIT2). All monoclonal antibodies were purchased from BD Bioscience (San Jose, CA, USA). Blood was stained in darkness for 30 minutes at 4°C and incubated with cell lysis buffer (BD Bioscience) for 10 minutes at room temperature. Then, cells were washed twice in PBS 0.01 M pH 7.4 and gently re-suspended. Samples were acquired and analyzed in a FACS Canto II with FACSDiva software (BD Bioscience). At least 5×10^4^ cells were acquired in the lymphocyte population gate according to their forward scatter (FSC) versus side scatter (SCC) features. Dead cells were excluded by light scatter (FSC-H versus FSH-A). All data shown for CD4+/CD8+ DP T cells are based on the CD3+ gate.

### Staining for intracellular markers

Analysis was done with 2×10^6^ of peripheral blood mononuclear cells (PBMC) from each individual, obtained by ficoll-hypaque density gradient (Sigma, St. Louis, MO, USA). Viability was assessed by trypan blue exclusion (Sigma). Surface staining was done with antibodies against CD3 APC, CD4 PerCP and CD8 PE-Cy7 for 30 minutes at 4°C. After one wash, the cell membrane was permeated with Cytofix/Cytoperm solution for 20 minutes at 4°C, followed by washing with Perm/Wash 1× (BD Bioscience). Intracellular staining was done with anti-perforin FITC antibody (clone δG9) or mouse IgG2b isotype control (clone 27–35) for 30 min at 4°C. Lastly, cells were washed, re-suspended and analyzed as described above. At least 2×10^5^ CD3+ cells were recorded by flow cytometry and perforin expression was determined in CD4+/CD8+ gate.

### Class I antigen specific double positive cells

As HLA-A2 is one of the most common class I allele in Colombia [23], donors were typed for HLA-A2 and subtype for HLA-A*0201 by flow cytometry and SS-PCR as previously described [24], [25]. Tetramer analysis was only done in HLA-A*0201+ individuals, including: 13 chagasic donors (6 asymptomatic and 7 symptomatic) and 5 healthy controls. Cells were incubated with anti-CD3 APC, anti-CD4 PerCP, anti-CD8 PE-Cy7 and anti-HLA-A*0201 PE tetramers loaded either with *T. cruzi* or influenza virus derived epitopes. *T. cruzi* epitope or K1, is a 9 mer peptide (TLEEFSAKL) derived from N-terminal region (amino acids 4–11) of *T. cruzi* KMP-11 protein (Swiss-Prot accession number: Q9U6Z1) [24], [25]. Influenza virus peptide was a modified epitope derived from the viral matrix proteins (a.a. 58–66) denominated MP-Flu (GILGFVTTL) [25]. Tetramers were synthesized by the National Institute of Health (NIH) Tetramer Facility (Atlanta, USA). At least 2×10^5^ cells were analyzed by flow cytometry in the CD3+ gate and tetramer expression was determined in the CD4+/CD8+ gates.

### Cell stimulation, degranulation and IFN-γ production

PBMC from 5 asymptomatic (G0) and 6 symptomatic patients (two of each: G1, G2 and G3) were isolated using density gradient as described above. A total of 2×10^6^ PBMC were culture in the presence of anti-CD28 (1 µg/ml) and anti-CD49d (1 µg/ml) and one of the following conditions: medium alone, 10 µg/ml of K1 peptide or 3.7 µg/ml of *Staphylococcal* enterotoxin B (SEB). Cultures were incubated for 3 hours at 37°C with 5% of CO_2_, followed by an additional 6 hours of incubation in the presence of Brefeldin A 10 µg/ml (BD Pharmingen). For degranulation assays, anti-CD107a and CD107b FITC (clones H4A3 and H4B4, respectively) were added immediately following stimulation. For surface staining, antibodies for CD3-Pacific Blue (UCHT1), CD4-PeCy5.5 (SK3), CD8-APCH7 (SK1) and K1 PE tetramer were used. PBMC were washed once and then fixed/permeated using Cytofix/Cytoperm (BD Bioscience). Cells were washed twice with Perm/Wash 1× (BD Bioscience), stained with 10 µl anti-IFN-γ PE-Cy7 (clone B27), rewashed once and re-suspended in FACs Flow (BD Bioscience). Population was gated according viability and CD3 expression. Expression of CD107 and production of IFN-γ was based on the K1 tetramer positive CD4+/CD8+ cells. At least 1×10^3^ tetramer positive cells were acquired and analyzed in a FACS Aria with FACSDiva software (BD Bioscience).

### Histology studies in cardiac tissue

Cardiac tissue from a chronic chagasic patient biopsy was used for immunochemistry. This specimen was obtained from a 47 year old male who was diagnosed with cardiac failure class III (NYHA classification) and underwent a cardiac transplant. Formalin-fixed and paraffin-embedded cardiac biopsy tissue was cut, deparaffinized in xylene and rehydrated with alcohol. Antigen retrieval was done heating the tissue in the presence of EDTA buffer pH 9.0. Slides were stained with hematoxylin/eosin to evaluate cellular infiltration and presence of parasites. Immunohistology was carried out with anti-human CD4 (clone 1F6, Vector Laboratories, Burlingame, CA, USA) and CD8 (C8/144B, DakoCytomation, Carpinteria, CA, USA) antibodies. After blocking endogenous enzymes, antibodies were revealed with EnVision™ G|2 Doublestain System kit (DakoCytomation) using peroxidase/DAB+ Chromogen for CD8 and then alkaline phosphatase/Permanent Red Chromogen for CD4. Slides were evaluated in a light microscope (40–100× objective magnification) where CD8+ cells yielded a brown-color and CD4+ a red-color end product; DP T cells showed a red-brown color.

### Statistical analysis

Descriptive statistics (mean, standard deviation and percentages) was used to describe the populations and to present the flow cytometry data. Non-parametric analysis was carried out (Statistix 8.0 software) for groups comparisons by Kruskal Wallis (K-W) test followed by Dunn post hoc tests. Comparison of two groups was done by Mann-Whitney U test (M-W). Significance was considered a *p* value<0.05.

## Results

### Phenotype analysis of double positive T cells

**Figure 1:**
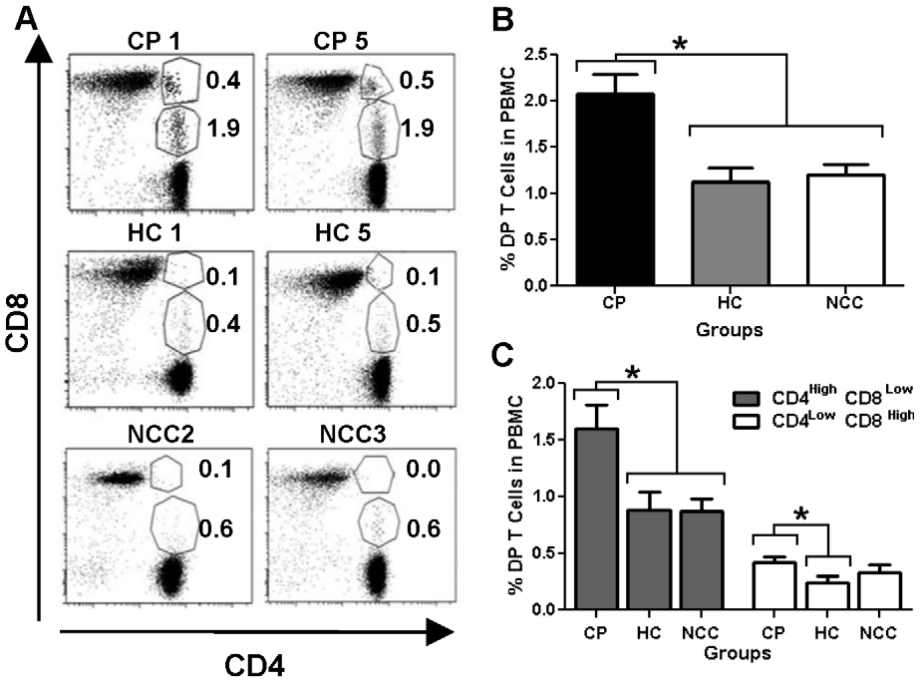
Percentage of circulating DP T cells in Chagasic patients and uninfected controls. (**A**) Representative dot plots of flow cytometry and gating strategy showing CD4+/CD8+ T cells subsets (CD4^high^CD8^low^ and CD4^low^CD8^high^) in chagasic patients (CP), healthy controls (HC) and non-chagasic cardiomyopathy (NCC) groups. DP T population was gated on lymphocytes (FSC versus SSC), viability and CD3 positive cells. (**B**) Averaged percentage with standard deviations (SD) of total CD4+/CD8+ T cells in PBMC from CP (black bars), HC (gray bars) and NCC (empty bars). (**C**) Comparison of averaged percentage of CD4^high^/CD8^low^ (gray bars) or CD4^low^/CD8^high^ (empty bars) T subsets and their standard deviations. *p<0.05 by Kruskal Wallis.

Cell frequency between chronic chagasic patients (CP) and healthy controls (HC) matched by age and gender were compared. The average percentage of total circulating DP T cells was higher (*p* = 0.0017) in CP (*X* = 2.1%±0.9) compared with HC group (1.1±0.5) (Figure 1B). Representative flow cytometry dot plots and gating strategy are shown in Figure 1A. To determine if this difference was attributed to the chagasic patients immune response and not to the cardiac damage or its pathological consequences, DP T cells percentages from CP were compared with those from donors with non-chagasic cardiomyopathy (NCC) (Figure 1A and 1B). Likewise, DP T cells frequency from CP was higher (*p* = 0.034) compared with NCC group (*X* = 1.2±0.4). For the following analysis, DP T cells were divided according to their phenotype into CD4^high^/CD8^low^ and CD4^low^/CD8^high^. The frequency of CD4^high^/CD8^low^ T cell was higher (*p* = 0.0058) in CP (*X* = 1.61%±0.87) than in HC (0.88±0.56) and NCC (0.87±0.41) donors (Figure 1C). Regarding to CD4^low^/CD8^high^ T cell, significant differences were only observed between CP and HC (*p* = 0.015) frequencies (Figure 1A and 1C).

**Figure 2:**
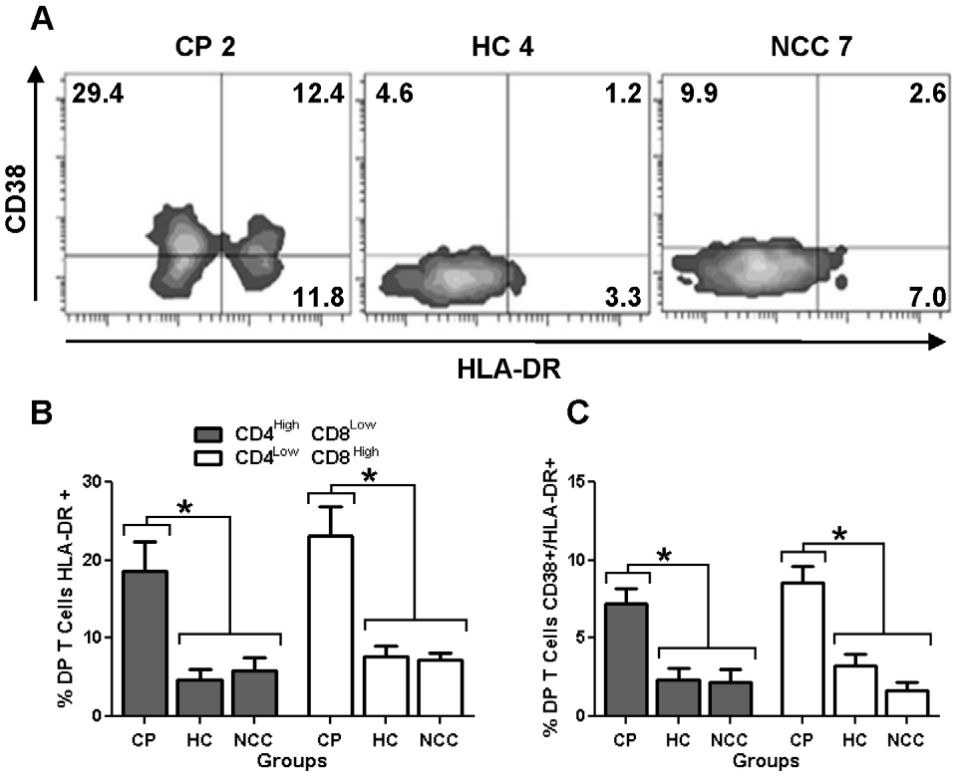
Activation markers in DP T cells from Chagasic patients and uninfected controls. (**A**) Representative density plots of CD38 and HLA-DR expression on DP T cells gated as indicated in Figure 1A from chagasic patients (CP), healthy controls (HC) and non-chagasic cardiomyopathy (NCC) donors. The percentage of CD38 and HLA-DR positive cells is shown in each quadrant. (**B**) Averaged percentage of HLA-DR+ events in CD4^high^/CD8^low^ (gray bars) or CD4^low^/CD8^high^ (empty bars) T cells. (**C**) Co-expression of CD38 and HLA-DR in CD4^high^/CD8^low^ (gray bars) or CD4^low^/CD8^high^ (empty bars) T cells and their standard deviations. *p<0.05 by Kruskal Wallis.

To define the activation status of DP T cells in chronic Chagas patients, CD38 and HLA-DR surface markers were analyzed. Since, it was previously reported that CD4^high^/CD8^low^ and CD4^low^/CD8^high^ T cells differed in the expression of several surface markers, phenotypic analysis was done separately [1]–[4], [10]. The percentage of DP T cells expressing HLA-DR was three times higher in CP in both CD4^high^/CD8^low^ (*p* = 0.0031) and CD4^low^/CD8^high^ (*p* = 0.01) populations than in the two control groups. No differences were observed between HC and NCC control groups (Figure 2B). Representative flow cytometry density plots and percentages of HLA-DR positive DP T cells are shown in Figure 2A and Figure 2B, respectively. Analysis of CD38 in DP T cells showed that CD4^high^/CD8^low^ subset had increased (*p* = 0.002) percentage of cell expressing this marker in CP (*X* = 38.9%±22.0) compared with NCC (9.6±5.3), and not different with HC (20.6±10.6). In contrast, the frequency of CD4^low^/CD8^high^ expressing CD38 was higher in CP (*X* = 30.0%±10.5) (*p* = 0.0001) when compared with both HC (12.6±7.7) and NCC (11.0±10.5). When co-expression of both activation markers (CD38 and HLA-DR) was analyzed, CP doubled the percentage of activated cells compared to HC and NCC donors in both CD4^high^/CD8^low^ (*p* = 0.009) and CD4^low^/CD8^high^ T cells (*p* = 0.001) (Figure 2A and 2C). There were no differences when the analysis by clinical status was done. Due to the similarity of both subsets of DP T cells (Table S1), data is presented on the whole population in subsequent analyses.

### Specificity and functional analysis of double positive T cells

**Figure 3:**
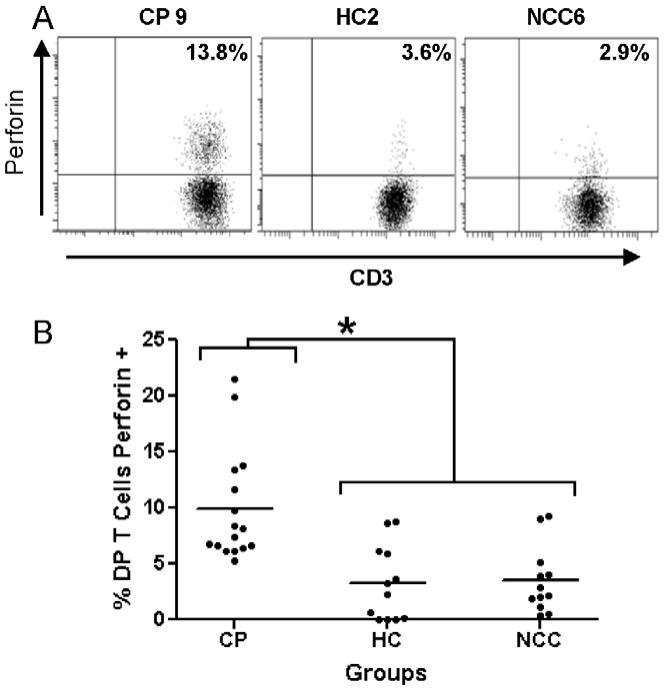
Functional characterization of DP T cells from Chagasic patients and uninfected controls. (**A**) Representative dot plot of perforin expression by CD4+/CD8+ T cells in chagasic patients (CP), healthy controls (HC) and non-chagasic cardiomyopathy (NCC) donors. DP T cells were selected as indicated in Figure 1A and the percentage of perforin positive cell is shown in each quadrant. (**B**) Percentages and average (line) of CD4+/CD8+ DP T cells expressing perforin in CP, HC and NCC donors. *p<0.05 by Kruskal Wallis.

To assess the cytotoxic potential of DP T cells, intracellular perforin was measured [10]–[11]. Increased (*p* = 0.002) percentages of cells containing perforin in CP (9.9±4.8) were found when compared with HC (3.3±3.2) and NCC (3.3±2.9) donors. Representative flow cytometry dot plots and percentage of perforin positive cells are shown in Figures 3A and 3B, respectively. No difference in perforin expression was found (*p* = 0.29) between asymptomatic (9.9±3.1) and symptomatic (9.9±5.7) chagasic patients.

**Figure 4:**
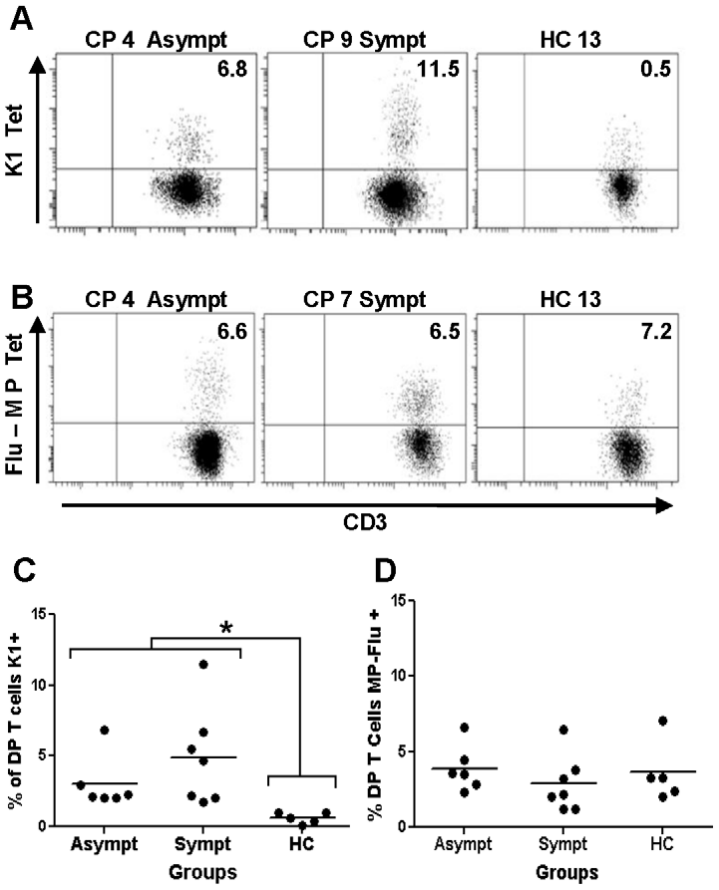
Antigen specificity of DP T cells from Chagasic patients and uninfected controls. (**A**) Representative dot plots DP T cells specific for K1 and (**B**) for MP-Flu tetramers in symptomatic CP (Sympt), asymptomatic CP (Asympt) donors and 5 healthy controls (HC). DP T cells were selected as indicated in Figure 1A and the percentage of tetramer positive cells in shown in each quadrant. Percentages and average (line) of DP T cells specific for K1 (**C**) and MP-Flu (**D**) peptides in chagasic patients and 5 uninfected donors (HC) typed for HLA-A*0201. *p<0.05 by Kruskal Wallis.

Next, the antigen specificity of DP T cells was determined. To do so, the frequency of HLA-A*0201/peptide recognition was assessed using epitopes from *T. cruzi* and influenza virus, denominated K1 (Figure 4A) and MP-Flu (Figure 4B), respectively [25]. We found that both asymptomatic (*X* = 3.0%±1.9) and symptomatic (4.9±3.6) CP had higher percentages (*p* = 0.0006) of circulating DP T cells specific for the K1 cytotoxic epitope than HC donors (0.6±0.4). There was no difference according to the disease stage (*p* = 0.267) (Figure 4C and Table S2). The percentage of K1-specific DP T cells in the Chagasic donors was in average 27 times higher (*p* = 0.0005) than the mono-CD8+ K1-specific ones (*X* = 4.0±2.9 versus *X* = 0.17±0.08, respectively), Table S2. Representative flow cytometry dot plots and percentage of K1 specific DP T cells are shown in Figure 4A and Figure 4C, respectively. Regarding MP-Flu response, asymptomatic (3.9±1.5), symptomatic (2.9±2.0) and HC (3.6±2.0) donors had similar percentages of specific DP T cells (*p* = 0.39) for this viral epitope. Representative flow cytometry dot plots and percentage of MP-Flu specific DP T cells are shown in Figures 4B and 4D, respectively.

**Figure 5:**
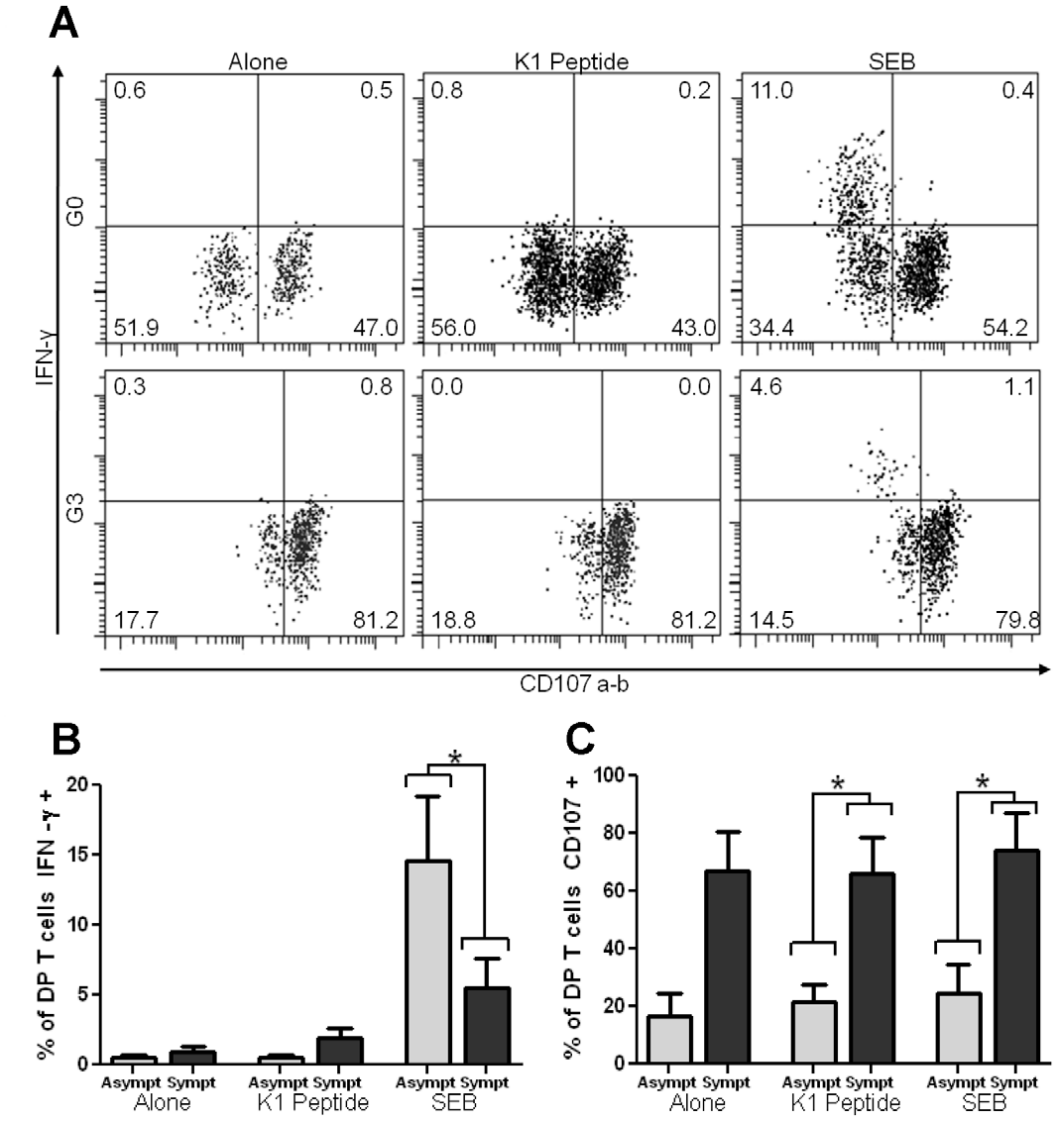
IFN-γ and CD107a/b expression by K1-tetramer positive DP T cells of Chagasic patients. (**A**) Representative dot plot of IFN-γ and CD107 expression by K1 Tet+/CD4+/CD8+ T cells in an asymptomatic (G0) and a symptomatic (G1–G3) chagasic patient. The percentage of IFN-γ and CD107 positive cells is shown in each quadrant. (**B**) Averaged percentage of IFN-γ positive K1 Tet +/CD4+/CD8+ T cells in asymptomatic (G0, grey bars) and symptomatic (G1–G3, black bars) chagasic patient. (**C**) Averaged percentage of CD107 positive K1 Tet+/CD4+/CD8+ T cells in asymptomatic (G0, grey bars) and symptomatic (G1–G3, black bars) chagasic patient. SEB, Staphylococcal enterotoxin B. *p<0.05 by Mann Whitney.

Production of IFN-γ and surface expression CD107 a/b, as marker of degranulation, were analyzed in K1 tetramer-positive DP T cells. The percentage of IFN-γ positive cells in medium alone was similar between asymptomatic (n = 5) (0.48%±0.45) and symptomatic patients (n = 6) (0.97%±0.97) (*p* = 0.52). Likewise, in the presence of K1 peptide, asymptomatic patients (1.87%±1.83) showed a no significant increase in the percentage of IFN-γ positive cells when compared with the symptomatic group (0.56%±0.43) (*p* = 0.26). However, after polyclonal stimulation (SEB), this percentage was significantly higher in the asymptomatic donors (14.6%±10.3 versus 5.55%±4.98; *p* = 0.049). Representative flow cytometry dot plots and percentage of IFN-γ positive cells are shown in Figures 5A and 5B, respectively. Furthermore, degranulation in K1 tetramer-positive DP T cells was significantly higher in than symptomatic donors in the presence of K1 peptide (21.50%±13.38 versus 66.25%±30.78; *p* = 0.0455) and SEB (24.74%±21.74 versus 74.20%±31.59; *p* = 0.03); this seemed to be also the trend for cell cultured in medium alone (16.58%±17.52 versus 67.0%±32.93; *p* = 0.052), in spite that no significant difference between the asymptomatic and symptomatic groups was found. Representative flow cytometry dot plots and percentage of CD107a/b positive cells are shown in Figures 5A and 5C, respectively.

### Histology studies in cardiac tissue

**Figure 6:**
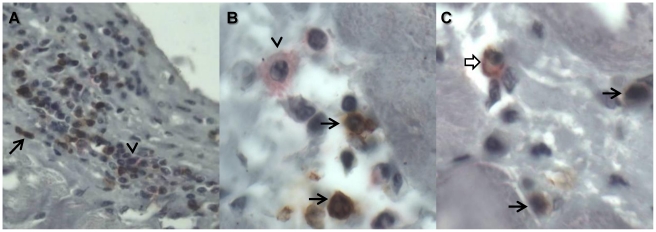
Staining for CD4 and CD8 in a chronic chagasic heart tissue. Immunochemistry for CD4 and CD8 staining in heart tissue from a chronic chagasic donor with cardiomyopathy. Slides were colored with hematoxyline/eosin to evaluate cellular infiltration and the presence of parasites. CD8+ cells yield a brown-color (arrows) and CD4+ a red-color end products (arrows head); DP T cells showed a red-brown color (wide arrow). (**A**) 40× magnification, and (**B**) and (**C**) 100× objective magnification.

Cardiac tissue from a chronic chagasic patient was analyzed in order to identify the presence of CD4+/CD8+ cells. The immunohistology analysis showed that myocardiocytes presented reparative nuclear changes such as bigger size, hyperchromatic and visible nucleoli. Moderate patchy myocardial infiltration mostly conformed by lymphocytes, some plasmocytes, macrophages and scarce eosinophils was also observed. The number of infiltrating lymphocytes was 10 or 12 per high power field, and they were mostly CD8+ cells (near 95%, Figure 6A and 6B) and some CD4+ cells (Figure 6B). Double CD4+/CD8+ cells were found in the inflammatory infiltrate of the cardiac tissue in a frequency lower than 2%, as shown in Figure 6C.

## Discussion

Circulating CD4+/CD8+ double positive (DP) T cells represent between 1–3% of the total T lymphocytes population [1]. However, previous evidence has suggested that this frequency can increase during several inflammatory diseases [2]–[8]. In this study we demonstrated that the percentage of peripheral DP T cells was higher in chronic chagasic patients compared with control donors, including individuals with non-chagasic cardiomyopathy. This finding differs from previous studies in HVC and HIV infected patients in which virus infected and control donors did not have differences in DP T cells frequencies on peripheral blood [10], [26]. To the best of our knowledge, this research is the first report of an augmented percentage of DP T cell in human patients with a chronic parasitic infection.

Previous characterization of DP T cells indicates that this lymphocyte subpopulation is constituted mainly by terminally differentiated memory cells. A recent study showed that chronic chagasic patients had higher frequencies of CD4+ effectors memory T cells (T_EM_) and CD8+ central memory T cells (T_CM_) when compared with uninfected individuals [27]. Interestingly, in healthy donors CD4^high^/CD8^low^ T cells were described as being T_EM_ phenotype, meanwhile CD4^low^/CD8^high^ were mainly T_CM_ [10]. It will be interesting to determine if some of those CD4+ T_EM_ and CD8+ T_CM_ described in chagasic patients could include some DP T cells [27].

Similarly to DP T cells described in other human chronic infectious diseases with antigen persistence [10], [26], [28], [29], we found that the expression of activation markers in these cells were increased in the chagasic donors. Consistently, some studies in *T. cruzi* infected donors have shown that their T cells (CD3+, CD3+/CD4+ and CD3+/CD8+) tended to be more activated than cells from uninfected donors [30]–[32].

Regarding the source of the peripheral DP T cells, experimental data supports that they might either escape from the thymus [33] or represents over-stimulated mono-positive CD4+ or CD8+ T cells [34], [35]. Interestingly, some studies have shown severe CD4+CD8+ thymocytes depletion coexisting with 16-fold increase of these cells in the periphery (subcutaneous lymph node) after *T. cruzi* acute infection in mice [21], [36]. These findings support the “thymus escape” theory. However, expression of the “second” marker in mono-positive T lymphocytes as a consequence of antigenic over-stimulation seems also plausible in our study, given the consistent activation exhibited by the DP T cells [34]. More studies are needed to determine the origin of DP T cells in chagasic patients.

Perforin-mediated cytotoxicity has proved to be very important in the protection and pathogenesis of some parasitic infection, including cerebral malaria [37] and toxoplasmosis [38]. In animal models, there is evidence that implicates perforin expression with *T. cruzi* elimination during the acute disease [32], [37]. However, perforin has also been involved in tissue damage during chronic Chagas disease [39]–[41]. After *T. cruzi* infection, perforin- knockout mice had an increased parasite burden and increased number of IFN-γ producing T cells infiltrating their hearts. However, these perforin-deficient animals showed more preserved cardiac tissue and less electric conduction abnormalities than normal littermates [39]. Even more, it has been suggested that human cardiac damage is directly related to an increase in the ratio of perforin-positive/total inflammatory cells in heart tissue [40].

Another remarkable result in this study is that DP T cells can recognized a *T. cruzi* derived class I epitope during chronic infection. The percentage of DP T cells specific for the K1 peptide was exceptionally high (1.7% to 11.5%) compared with previous reports of K1 specificity for mono-CD8+ T lymphocytes (0.09% to 0.34%) in a similar chronic chagasic population [25]. Likewise, in human viral infections (HCV and HIV) upon antigen exposure, DP T cells displayed a much higher frequency of cell-single cytokine production than CD8+ and CD4+ T cells [10], [26]. Analysis of class I antigen recognition suggests that DP T cells in chagasic patients were parasite driven. Probably, as these peripheral cells are *T. cruzi* antigen-specific and have memory phenotype, they could migrate to the tissue where parasites persist and contribute to *T. cruzi* induced pathology. In fact, in situ cytotoxic lymphocytes (perforin or granzyme A positive cells) have been described in human heart [41] and gastrointestinal tract [42] of patients with *T. cruzi* induced tissue damage.

When activation markers, perforin and K1-recognition on DP T cells were compared in chagasic patients according to their clinical status (asymptomatic versus symptomatic patients), no differences were found. Nevertheless, it was notable that some symptomatic donors had higher percentages of K1 *T. cruzi* specific cells than asymptomatic ones, while percentages of recognitions for influenza virus epitope were similar. We also found that K1 specific DP T cells from symptomatic group displayed increased degranulation activity even with medium alone. However, very low percentages of these K1 tetramer specific DP T cells produced IFN-γ after K1 peptide stimulation, as similarly described for CD8+ T cells in chronic chagasic patients [25]. This was not the case for the production of IFN-γ after polyclonal stimulation, which was significantly augmented especially in the asymptomatic patients. Interestingly, K1 specific DP T cells that produce IFN-γ did not display degranulating phenotype, indicating that DP T population is functionally heterogeneous and complex. In summary, K1 specific cells from asymptomatic donors had higher capacity of IFN-γ secretion than cells from symptomatic donors which have greater cytotoxic potential.

In our study, we found a higher expression of perforin on peripheral DP T cells in the chagasic patients accompanied with an increased degranulation activity in the symptomatic ones. Also, it was demonstrated that DP T cells can migrated to the cardiac tissue. If the blood phenotype of these cells is maintained by the infiltrating ones, it might be possible to associate DP T cells with cardiac damage. A similar mechanism was suggested for HCV infected humans where DP T cells were found to infiltrate the liver [10].

Progressive loss of cytokines secretion is a characteristic of CD8+ T cells exhaustion, a phenomenon related to antigen persistence during chronic infections [43]. Indeed, IFN-γ that is associated with protection against *T. cruzi* infection [44], is one of the last effector activities to be extinguished in this process [43]. So, our data suggest that K1 specific DP T cells, mainly from symptomatic donors, should be on the pathway of exhaustion, while they keep their cytotoxic potential. Lastly, as TNFα production has been associated with cardiac damage in Chagas disease [45], it will be of interest to test the production of TNFα on *T. cruzi* specific DP T cells.

## Supporting Information

Table S1 **Comparison of DP T cells among chagasic donors according to the presence of symptoms.** Modified Kuschnir Classification: Asymptomatic (G0) and symptomatic (G1, G2 and G3).
(DOC)

Table S2 **Percentages of K1-tetramer positive DP T cells and mono-CD8+ T cells from chagasic patients.** (**A**) K1 positive DP T cells frequencies in asymptomatic versus symptomatic patients, *p* = 0.267 (Mann Whitney). (**B**) K1 positive CD8+ T cells frequencies in asymptomatic versus symptomatic patients, *p* = 0.798. (**C**) K1 positive DP T cell versus with CD8+ T cells frequencies, *p* = 0.0005.
(DOC)
