# Supplementary material for: Increased CD4+/CD8+ Double-Positive T Cells in Chronic Chagasic Patients
Source: PLoS Negl Trop Dis. 2011 Aug 23;5(8):e1294. doi: 10.1371/journal.pntd.0001294 (PMC3160296; doi:10.1371/journal.pntd.0001294)
Supplement: Table S1 — Comparison of DP T cells among chagasic donors according to the presence of symptoms. Modified Kuschnir Classification: Asymptomatic (G0) and symptomatic (G1, G2 and G3). (DOC) [file pntd.0001294.s001.doc]

**Table S1. Comparison of DP T cells among chagasic donors according to the presence of symptoms.**

|  | Symptomatic | Asymptomatic | P value |
| --- | --- | --- | --- |
| CD4^High^/CD8^Low^ | 1.7 ± 0.9 | 1.5 ± 0.9 | 0.4788 |
| CD38+/HLA DR+ | 5.9 ± 4.0 | 8.1 ± 4.4 | 0.4126 |
|  |  |  |  |
| CD4^High^/CD8^Low^ | 0.4 ± 0.3 | 0.4 ± 0.1 | 0.0548 |
| CD38+/HLA DR+ | 7.7 ± 3.7 | 9.2 ± 4.5 | 0.3240 |
|  |  |  |  |
| CD4+/CD8+ Total | 2.2 ± 0.8 | 2.0 ± 0.9 | 0.3872 |
| Perforin + | 9.9 ± 3.1 | 9.8 ± 5.6 | 0.2895 |
| Tet K1 + | 3.0 ± 1.9 | 4.9 ± 3.6 | 0.2638 |
| Tet Flu + | 3.9 ± 1.5 | 2.9 ± 2.0 | 0.3902 |
