# Supplementary material for: Increased CD4+/CD8+ Double-Positive T Cells in Chronic Chagasic Patients
Source: PLoS Negl Trop Dis. 2011 Aug 23;5(8):e1294. doi: 10.1371/journal.pntd.0001294 (PMC3160296; doi:10.1371/journal.pntd.0001294)
Supplement: Table S2 — Percentages of K1-tetramer positive DP T cells and mono-CD8+ T cells from chagasic patients. (A) K1 positive DP T cells frequencies in asymptomatic versus symptomatic patients, p = 0.267 (Mann Whitney). (B) K1 positive CD8+ T cells frequencies in asymptomatic versus symptomatic patients, p = 0.798. (C) K1 positive DP T cell versus with CD8+ T cells frequencies, p = 0.0005. (DOC) [file pntd.0001294.s002.doc]

**Table S2**. **Percentages of K1-tetramer positive DP T cells and mono-CD8+ T cells from chagasic patients.**

|  |  | % K1 tetramer cells | | |
| --- | --- | --- | --- | --- |
|  | Disease stage | DPTC^a^ | CD8^b^ | DPTC/CD8 |
| Asymptomatic | G0 | 2,1 | 0,23 | 9,1 |
|  |  | 6,8 | 0,15 | 45,3 |
|  |  | 2,1 | 0,05 | 42,0 |
|  |  | 2,0 | 0,34 | 5,9 |
|  |  | 2,3 | 0,09 | 25,6 |
|  |  | 2,9 | 0,13 | 22,3 |
|  | Mean | 3,0 | 0,17 | 25,0 |
|  | SD | 1,9 | 0,11 | 16,3 |
| Symptomatic | G1 | 5,5 | 0,17 | 32,4 |
|  |  | 6,7 | 0,12 | 55,8 |
|  | G2 | 4,7 | 0,29 | 16,2 |
|  |  | 11,5 | 0,17 | 67,6 |
|  |  | 2,2 | 0,22 | 10,0 |
|  | G3 | 1,7 | 0,14 | 12,1 |
|  |  | 2,0 | 0,13 | 15,4 |
|  | Mean | 4,9 | 0,18 | 29,9 |
|  | SD | 3,5 | 0,06 | 23,1 |
| Total | Mean^c^ | 4,0 | 0,17 | 27,7 |
|  | SD | 2,9 | 0,08 | 19,6 |
